# Detection, Prevalence and Phylogenetic Relationships of *Demodex* spp and further Skin Prostigmata Mites (Acari, Arachnida) in Wild and Domestic Mammals

**DOI:** 10.1371/journal.pone.0165765

**Published:** 2016-11-01

**Authors:** Natalia Sastre, Olga Francino, Joseph N. Curti, Tiffany C. Armenta, Devaughn L. Fraser, Rochelle M. Kelly, Erin Hunt, Katja Silbermayr, Christine Zewe, Armand Sánchez, Lluís Ferrer

**Affiliations:** 1 Department of Ecology and Evolutionary Biology, University of California, Los Angeles, California, United States of America; 2 Servei Veterinari de Genètica Molecular, Universitat Autònoma de Barcelona, Bellaterra, Spain; 3 The Rocky Mountain Biological Laboratory, Crested Butte, Colorado, United States of America; 4 Department of Biology, University of Washington, Seattle, Washington, United States of America; 5 California Wolf Center, Julian, California, United States of America; 6 Department of Pathobiology, Institute of Parasitology, Vetmeduni Vienna, Vienna, Austria; 7 Department of Clinical Sciences, Cummings School of Veterinary Medicine, Tufts University, North Grafton, Massachusetts, United States of America; University of the Sunshine Coast, AUSTRALIA

## Abstract

This study was conceived to detect skin mites in social mammals through real-time qPCR, and to estimate taxonomic *Demodex* and further Prostigmata mite relationships in different host species by comparing sequences from two genes: mitochondrial *16S rRNA* and nuclear *18S rRNA*. We determined the mite prevalence in the hair follicles of marmots (13%) and bats (17%). The high prevalence found in marmots and bats by sampling only one site on the body may indicate that mites are common inhabitants of their skin. Since we found three different mites (*Neuchelacheles* sp, *Myobia* sp and *Penthaleus* sp) in three bat species (*Miotis yumanensis*, *Miotis californicus* and *Corynorhinus townsendii*) and two different mites (both inferred to be members of the Prostigmata order) in one marmot species (*Marmota flaviventris*), we tentatively concluded that these skin mites 1) cannot be assigned to the same genus based only on a common host, and 2) seem to evolve according to the specific habitat and/or specific hair and sebaceous gland of the mammalian host. Moreover, two *M*. *yumanensis* bats harbored identical *Neuchelacheles* mites, indicating the possibility of interspecific cross-infection within a colony. However, some skin mites species are less restricted by host species than previously thought. Specifically, *Demodex canis* seems to be more transmissible across species than other skin mites. *D*. *canis* have been found mostly in dogs but also in cats and captive bats. In addition, we report the first case of *D*. *canis* infestation in a domestic ferret (*Mustela putorius*). All these mammalian hosts are related to human activities, and *D*. *canis* evolution may be a consequence of this relationship. The monophyletic *Demodex* clade showing closely related dog and human *Demodex* sequences also supports this likely hypothesis.

## Introduction

*Demodex* mites are arthropods that belong to the class Arachnida and subclass Acari. Since Simon described the first *Demodex* in 1942, over 140 *Demodex* species have been identified in at least 11 orders of domestic and wild mammals [1–3]. Most mammals, including humans harbor *Demodex* mites on the skin without developing lesions or any other clinical signs [4–6]. However, changes in the host’s cutaneous environment and immune response can lead to mite overgrowth, lesions and other clinical signs [1,7]. *Demodex* proliferation in hair follicles and glands, such as sebaceous, Meibomian and/or ceruminous glands, can cause a severe and prevalent dermatitis in the host [1,2,8]. Canine and feline demodicosis is a well-known example of severe dermatitis caused by the proliferation of *Demodex* mites [9]. In humans, an overabundance of *Demodex* mites has been associated with rosacea, a facial skin disorder that affects well over 16 million Americans [10] (National Rosacea Society-Rosacea.org), although the role played by the mites in the pathogenesis of the disease remains unclear.

In addition to its medical importance, the study of *Demodex* parasites is of great interest for evolutionary biology. The widespread occurrence of skin mites throughout the mammalian class suggests that the parasitic relationship is very ancient and may have been established soon after the initial radiation of mammals 220 million years ago, when animals with hair follicles first appeared [11,12]. Approximately 100 million years ago, the clade Boreoeutheria split into two sister taxa: Euarchontoglires, including primates and rodents, and Laurasiatheria, constituted mainly by hoofed and carnivorous mammals, including cervids, mustelids, canids and felids but also chiroptera [13]. *Demodex* mites have been described (both morphologically and genetically) in both groups, reflecting the constancy of the hair follicle niche or the high transmissibility of *Demodex* mites between and within placental mammal species [5,14–20]. We have an extraordinary example of parasitic parallelism in the *Demodex* genus [6,12,21,22].

Historically, *Demodex* mite classification has been primarily based on sharing a common mammalian host and morphological features. For instance, species pairs *D*. *folliculorum* and *D*. *brevis* found on humans [23], *D*. *canis* and *D*. *injai* found on dogs [24–26] and *D*. *cati*, *D*. *gatoi* and *D*. *felis* found on cats [27–29] are assumed to be sister groups. More recently, genetic approaches have been used to establish phylogenetic relationships within the family Demodicidae. Specifically, randomly amplified polymorphic DNA (RAPD) has been used to analyze mite variability and relationships [30,31]. In addition, a 930 bp fragment of the mitochondrial *Demodex* genome was used to evaluate the genetic diversity and phylogeography of *D*. *folliculorum* [21]. However, one of the most effective genes known to detect, determine and classify *Demodex* mites is the mitochondrial *16S rRNA* gene (16S rDNA). The taxonomic relationships of *Demodex* mites have also been estimated using the nuclear *18S rRNA* gene [19,22]. While the *16S rRNA* gene provides more discrimination at lower taxonomic levels [14,18,20,32,33], the *18S rRNA* gene is considered more appropriate for classifications at the level of phyla and superphyla [34,35].

Acari form seven orders, four of which are parasitic: Metastigmata (ticks) and Mesostigmata, Astigmata and Prostigmata (mites). Prostigmata (= Trombidiformes) implies one or two pairs of stigmata in the region of the gnathosoma associated with the chelicerae (commonly referred to as “jaws”) [36]. Prostigmata is one of the most diverse orders within Acariformes. Several morphological and ecological differences reflect this diversity. The feeding ranges from phytophagous to fungivorous, algivorous and saprophagous, and the parasitic habitats affect both vertebrate and invertebrate hosts [36,37]. Prostigmata comprises two large clades, Eupodina (that includes Labidostommatina) and Anystina (that includes Eleutherengona) [38]. The cohort Eleutherengona comprises four superfamilies: Heterostigmata and Cheyletoidea and Tetranychoidea as sister groups, with Raphignathoidea as the sister group to the lineage giving rise to Cheyletoidea and Tetranychoidea [38]. *Demodex* mites belong to the superfamily Cheyletoidea and family Demodecidae. Finally, the Eupodina clade comprises six superfamilies: Bdelloida and Halacaroidea as sister groups with Labidostommatoidea as the sister group to the lineage and Eriophyoidea and Tydeoidea as sister groups with Eupodoidea [38].

The first aim of this investigation was to determine the prevalence of mites (using 16S rDNA primers that successfully amplified clinical *Demodex* samples [5,18,32,39]) in both captive and free-ranging wild mammals, including gray wolves (*Canis lupus*), yellow-bellied marmots (*Marmota flaviventris*), and four species of vespertillionid bats (*Myotis californicus*, *M*. *lucifugus*, *M*. *yumanenses* and *Corynorhinus townsendii*) distributed throughout North America. We chose these particular species because of their social behavior. Since physical contact among host individuals promotes parasite transmission, we expected high mite prevalence in colonial or social animals [40]. In the wild, wolves maintain a defined social hierarchy, which revolves around the reproductive pair [41]. A single wolf pack consists of a breeding pair and their offspring of the previous one to three years, while in large wolf packs multiple litters can coexist [42]. In contrast, wolves in captivity are often held in enclosures with both close-relatives and strangers. Yellow-bellied marmots are hibernating ground-dwelling squirrels that inhabit subalpine meadows, slopes and clearings [43]. Marmots are social mammals, with colony sizes ranging from one adult male, one adult female and their offspring to large colonies with several breeding females, sometimes including as many as thirty individuals [43,44]. Vespertillionid bats are widely distributed across North America, with summer maternity colony sizes ranging from a few dozen for *M*. *californicus* to upwards of several thousand individuals for *M*. *yumanensis* and hundreds of thousands for *M*. *lucifugus* [45,46]. *Myotis* spp. frequently forage near or over water (e.g. streams, ponds and lakes). *Corynorhinus townsendii* are distributed primarily in the western parts of US, Canada and Mexico. Summer maternity colony sizes for *C*. *townsendii* rarely reach above 100 individuals and colonies are typically in caves, abandoned mines, hollow trees and abandoned buildings [45]. Because group size has been shown to predict parasite risk in mammals, we expect skin mites to be prevalent within these populations [40].

The second aim was to determine the genetic variability of *Demodex* spp by comparing sequences from two genes: mitochondrial *16S rRNA* and nuclear *18S rRNA*. *Demodex* mites were additionally isolated from domestic dogs (*Canis familiaris*), cats (*Felis catus*) and one ferret (*Mustela putorius*) showing clinical signs and diagnosed with generalized demodicosis. Moreover, we isolated a *Demodex folliculorum* from a healthy human (*Homo sapiens*). The results were then used to establish the phylogenetic relationships within mites in social, wild, healthy mammals and unhealthy domestic mammals.

## Material and Methods

### Ethics Statement

Hair samples from captive gray wolves were collected from the California Wolf Center (CWC) (Julian, California, USA;”33.03236 Latidude, -116.54584 Longitude”), a 501(c)(3) non-profit center dedicated to the recovery of wolves in the wildlands they once roamed. Hair samples were collected in conjunction with annual health checks which CWC staff conducts on each wolf in January to provide vaccines, perform blood and fecal tests and assess overall physical condition. Hair samples from wild yellow-bellied marmots (*Marmota flaviventris*) were collected in the East River Valley around the Rocky Mountain Biological Laboratory (RMBL) in Gunnison County (Colorado, USA;”38.98831, -107.00979”). Marmots are not protected and they were trapped on publicly owned lands. Once hair samples were extracted, marmots were immediately released unharmed back into their environment. All procedures were approved under research protocol ARC 2001-191-01 as well as permits issued by the Colorado Division of Wildlife. The research protocol was approved by the University of California Los Angeles Animal Care Committee on 13 May 2002 and renewed annually. Hair samples were obtained from vespertillionid bats captured on the San Juan Islands (Washington, USA; “48.55137, -123.07811”), Douglas County (Washington, USA; “47.77906, -119.74746) and Whatcom County (Washington, USA; “48.87872, -121.97187”). Bats are not protected and they were trapped on publicly owned lands. Once hair samples were extracted, bats were immediately released unharmed back into their environment. All procedures were approved by the University of Washington and permits by the Washington department of Fish and Wildlife (IACUC number 4307–01). Hair samples from domestic animals were plucked from one of the co-authors and dogs, cats and a ferret affected by demodicosis. Owners gave their consent for participating in this study. Animals were not harmed in any way during the hair collection.

### Sampling

Twenty-two hair samples from healthy captive gray wolves were collected from the CWC. The CWC keeps fourteen Mexican wolves (*Canis lupus baileyi*) in four different enclosures and eight Alaskan wolves (*Canis lupus occidentalis*) in a separate enclosure (no wolf remains alone).

Sixteen hair samples from wild yellow-bellied marmots (*Marmota flaviventris*) were plucked from the rumps of healthy marmots living in the East River Valley around the RMBL in Gunnison County (Colorado, USA). Six sites were sampled corresponding to six different colonies (Table 1). All sampled individuals appeared to be healthy at the time of capture (ranging from May to September 2014), there were no known diseases in the population at the time and no abnormalities were observed.

**Table 1:** Sample collection.

Host	Sample	Area	Coll. Year	Collection Place	16S qPCR Detection	GenBank sequence number 16S	GenBank sequence number 18S	Tree name
Bat A^a^	B01	Skin	2014	Site 6, WA^i^-US	**Positive**	**KT259444**	**KU253783**	mite_bat1UAB
Bat A	B02	Skin	2014	Site 6, WA-US	**Uncertain**	x	x	
Bat A	B03	Skin	2014	Site 6, WA-US	Negative			
Bat A	B04	Skin	2014	Site 6, WA-US	Negative			
Bat A	B05	Skin	2014	Site 6, WA-US	Negative			
Bat A	B06	Skin	2014	Site 6, WA-US	Negative			
Bat A	B07	Skin	2014	Site 6, WA-US	Negative			
Bat A	B08	Skin	2014	Site 6, WA-US	Negative			
Bat A	B09	Skin	2014	Site 6, WA-US	Negative			
Bat A	B10	Skin	2014	Site 8, WA-US	Negative			
Bat B	B11	Skin	2014	Site 8, WA-US	**Positive**	**KU253782**	**KU253785**	mite_bat3UAB
Bat A	B12	Skin	2014	Site 8, WA-US	**Positive**	**KT259444**	**KU253783**	mite_bat1UAB
Bat A	B13	Skin	2014	Site 8, WA-US	Negative			
Bat B	B14	Skin	2014	Site 7, WA-US	Negative			
Bat A	B15	Skin	2014	Site 6, WA-US	Negative			
Bat A	B16	Skin	2014	Site 6, WA-US	**Positive**	**KT259444**	**KU253783**	mite_bat1UAB
Bat A	B17	Skin	2014	Site 6, WA-US	Negative			
Bat A	B18	Skin	2014	Site 6, WA-US	Negative			
Bat D^d^	B19	Skin	2014	Site 12, WA-US	**Positive**	**KT259445**	**KU253784**	mite_bat2UAB
Bat D	B20	Skin	2014	Site 12, WA-US	Negative			
Bat A	B21	Skin	2014	Site 11, WA-US	Negative			
Bat B^b^	B22	Skin	2014	Site 10, WA- US	Negative			
Bat A	B23	Skin	2014	Site 10, WA- US	Negative			
Bat A	B24	Skin	2014	Site 10, WA- US	Negative			
Bat A	B25	Skin	2014	Site 9, WA-US	Negative			
Bat C^c^	B26	Skin	2014	Site 9, WA-US	Negative			
Bat A	B27	Skin	2014	Site 6, WA-US	Negative			
Bat A	B28	Skin	2014	Site 6, WA-US	Negative			
Marmot^e^	M01	Rump	2014	Col1^j^; CB^k^, CO^m^-US	Negative			
Marmot	M02	Rump	2014	Col2; CB, CO-US	**Positive**	**KT259446**	x	mite_marmot1UAB
Marmot	M03	Rump	2014	Col3; CB, CO-US	Negative			
Marmot	M04	Rump	2014	Col3; CB, CO-US	Negative			
Marmot	M05	Rump	2014	Col1; CB, CO-US	Negative			
Marmot	M06	Rump	2014	Col4; CB, CO-US	**Uncertain**	x	x	
Marmot	M07	Rump	2014	Col1; CB, CO-US	**Positive**	**KT259447**	x	mite_marmot2UAB
Marmot	M08	Rump	2014	Col5; CB, CO-US	Negative			
Marmot	M09	Rump	2014	Col1; CB, CO-US	Negative			
Marmot	M10	Rump	2014	Col4; CB, CO-US	Negative			
Marmot	M11	Rump	2014	Col4; CB, CO-US	Negative			
Marmot	M12	Rump	2014	Col4; CB, CO-US	Negative			
Marmot	M13	Rump	2014	Col1; CB, CO-US	Negative			
Marmot	M14	Rump	2010	Col1; CB, CO-US	**Uncertain**	x	x	
Marmot	M15	Rump	2010	Col1; CB, CO-US	Negative			
Marmot	M16	Rump	2014	Col6; CB, CO-US	Negative			
Dog^f^	D01	Leg	2015	MA^n^-US	-	JX390978	**KU253790**	D. canisUAB
Dog	D02	Leg	2015	MA-US	-	JF784000	**KU253790**	D. canis1/D. canisUAB
Dog	D03	Face/Dorsum	2015	RI^o^-US	-	**KT259448**	**KU253790**	D. dog3/D. canisUAB
Dog	D04	Dorsum	2015	MA-US	-	JX390978	**KU253790**	D. canisUAB
Dog	D05	Face/neck	2015	MA-US	-	JX390978	**KU253790**	D. canisUAB
Dog	D07	Thorax	2015	MA-US	-	JX390978	**KU253790**	D. canisUAB
Dog	D08	Thorax	2015	Grenada, East Caribbean	-	JX390978	**KU253790**	D. canisUAB
Dog	DMX149	Skin	2012	UAB^p^-Spain	-	JX390978	**KU253790**	D. canisUAB
Dog	DMX169	Leg	2014	Vienna, Austria	-	JX390978	**KU253790**	D. canisUAB
Dog	DMX154	Skin	2012	UAB-Spain	-	JX390979	**KU253790**	D. corneiUAB/D. canisUAB
Dog	D09	Dorsum	2015	MA-US	-	**KT259449**	**KU253789**	D. injaiUAB
Dog	DMX151	Skin	2012	UAB-Spain	-	JX390980	**KU253789**	D. injaiUAB
Dog	DMX168	Lumbar	2014	Vienna, Austria	-	JX390980	**KU253789**	D. injaiUAB
Cat^g^	DMX155	Neck	2013	Vienna, Austria	-	KF052996	**KU253786**	D. gatoiUAB
Cat	DMX156	Skin	2013	UAB-Spain	-	KF052995	**KU253787**	D. felisUAB
Cat	DMX166	Abdominal/Leg	2013	Vienna, Austria	-	JX193759	**KU253788**	D. catiUAB
Cat	DMX167	Face	2014	Korneuburg, Austria	-	JX193759	**KU253788**	D. catiUAB
Ferret^h^	D11	Leg	2015	MA-US	-	**KU253781**	**KU253791**	D. canisUAB
Human	DMX152	Face	2012	UAB-Spain	-	JF783995	KF745889	D. folliculorumUAB

^a^Bat A *(Myotis yumanensis)*

^b^Bat B (*Myotis californicus*)

^c^Bat C (*Myotis lucifugus*)

^d^Bat D *(Corynorhinus townsendii)*

^e^Marmot *(Marmota flaviventris)*

^f^Dog *(Canis familiaris)*

^g^Cat *(Felis catus*)

^h^Ferret *(Mustela putorius)*

^i^WA: Washington

^j^Col: colony

^k^CB: Crested Butte

^m^CO: Colorado

^n^MA: Massachusetts

^o^RI: Rhode Island

^p^UAB: University Autonomous of Barcelona. In bold, new sequences.

Twenty-eight hair samples were obtained from healthy vespertillionid bats captured on the San Juan Islands (Washington, USA) and on the eastern side of Washington State from June to September 2014. Twenty-two samples were collected from *M*. *yumanensis*, three samples were from *M*. *californicus*, one sample from *M*. *lucifugus* and the remaining two samples were from *Corynorhinus townsendii*. All bats were caught using a mist net at seven different sites (Table 1); however, the number of colonies could not be determined since we did not know where the captured bats roost.

All hair samples were collected with gloved hands or surgical mosquito forceps. Hair was plucked in the direction of growth to include the hair bulb (root) for DNA extraction. Hair samples were obtained from one site on the body in marmots and bats and five sites in wolves. The specific sampling areas of the wolf skin were based on the best areas to detect *Demodex* mites in dog skin [6,32]: face, dorsum, lumbar, abdomen and feet. In three wolves, we also plucked hair from injury/scab areas. In total, we obtained hair samples from 22 wolves (113 samples), 28 bats and 16 marmots.

*Demodex* mites from domestic animals were isolated individually from skin scrapings of a healthy human and thirteen dogs, four cats and a ferret, affected by demodicosis, at the Cummings School of Veterinary Medicine at Tufts University (Massachusetts, USA), at the Veterinary School, Universitat Autònoma de Barcelona (UAB) (Spain) and at the Institute of Parasitology, University of Veterinary Medicine, Vienna (Austria) (Table 1 and S1 Table). The mites were examined microscopically and morphologically, and identified as: *D*. *canis* and *D*. *canis* variant *cornei* (ten samples; host: dog), *D*. *injai* (three samples; host: dog), *D*. *cati* (two samples; host: cat), *D*. *gatoi* (one sample; host: cat), *D*. *felis* (one sample; host: cat), *D*. *folliculorum* (one sample; host: human) and *Demodex* spp similar to *D*. *canis* (one sample, from the ferret). All samples were stored at –20°C until DNA extraction.

### DNA extraction and real-time PCR (qPCR) amplification

DNA from hair bulb and skin scraping samples was extracted according to Ravera et al. [39]. Briefly, samples were centrifuged in a microcentrifuge at 16,000×*g* for 15 min. The pellet was re-suspended in 200 μl of digestion buffer (50 mM Tris–HCl, pH 8.5; 1 mM EDTA) and 10 μl of proteinase K solution (10 mg/ml, Roche Life Science). Samples were incubated at 56°C overnight. Then, the proteinase K was inactivated for 10 min at 95°C and the samples were centrifuged at 16,000*×g* for 15 min. Supernatant was diluted 1:5 for qPCR amplification.

Universal 16S primers, described by Ferreira et al. [5], were used to amplify an approximately 338-bp fragment of the mitochondrial *16S rRNA* gene. Hair samples from wolves, marmots and bats were amplified using real-time qPCR. Positive *Demodex* qPCR controls were obtained from isolated mite DNA that was previously amplified and sequenced. Blank DNA extractions and negative (UltraPure™ Distilled Water, Invitrogen) qPCR controls were also used to detect exogenous DNA contamination. We amplified each sample in duplicate. Real-time qPCRs were carried out under a laminar flow hood in a final volume of 15 μL using FastStart Universal SYBR Green Master (Roche Life Science), 0.5 μM of each primer and 5 μL of 1:5 diluted DNA. Thermal cycling qPCR profiles were 95°C for 10 min followed by 45 cycles at 95°C for 15 s and 60°C for 1 min. Samples were placed in a LightCycler® 480 Multiwell Plate 384 (Roche), and real-time qPCRs were performed in a Roche LightCycler 480 Real-Time PCR System (Roche Life Science). qPCR specificity assessment was performed by adding a Melting Curve analysis at the end of the run. Universal 16S qPCR products showing melting curves between Tm = 72°C and 82°C and amplification cycles between Cp = 25 cycles and Cp = 39 cycles were sequenced.

Isolated *Demodex* mites from thirteen domestic dogs, four cats, one human and one domestic ferret were amplified using the same primers described above through conventional PCR. We amplified a 338-bp DNA fragment of the mitochondrial *16S rRNA* gene. We used the PCR conditions described in Sastre et al. [18]. Briefly, PCR mixture was prepared in a 20μl reaction containing 5 μl of diluted DNA, PCR buffer (x1), 1.5 mmol⁄L MgCl2, 0.2 mmol⁄L of each dNTP, 0.5 μmol/L of each primer and 1 U AmpliTaq Gold DNA Polymerase (Life Technologies Corp.). The thermal cycling profile included 10 min at 94°C, followed by 35 cycles of 94°C (30 s), 57°C (30 s) and 72°C (30 s), and then completed with 10 min at 72°C.

For the *18S rRNA* gene, we used conventional PCR to amplify a fragment of approximately 530 bp from the samples that had been sequenced successfully for the *16S rRNA* gene. The primer pairs to amplify the fragment were as follows: 18S-F forward 5’-TCCAAGGAAGGCAGCAGGCA-3’ and 18S-R reverse 5’-CGCGGTAGTTCGTCTTGCGACG-3’. We used the same PCR conditions described above except for an increased annealing temperature of 60°C instead of 57°C.

All qPCR and PCR products were sequenced with BIG Dye^TM^ Terminator Cycle Sequencing Ready Reaction Kit, version 3.1 (Life Technologies Corp.) following the manufacturer’s protocol. Sequences were purified using the Montage SEQ96 Sequencing Reaction Cleanup Kit (Millipore, MA, USA) and separated on an ABI PRISM 3730 automated sequencer (Life Technologies Corp.) according to the protocol provided by the manufacturer.

### Genetic and phylogenetic analysis

The number of haplotypes (H), nucleotide diversity (*π)* and number of polymorphic sites (*S*) were estimated using DNASP 5.10 [47]. The nucleotide composition and pairwise genetic distances within and between populations were estimated with MEGA 6.06 [48]. Since *p*-distance may underestimate the true genetic distance because some of the nucleotide positions may have experienced multiple substitutions events, pairwise genetic distances were calculated using Kimura 2-parameter distances (K2P) [49]. All ambiguous positions were removed for each sequence pair.

For comparisons and phylogenetic analysis, we used the sequences of several Acari classes belonging to the orders Sarcoptiformes and Trombidiformes published in GenBank. All sequences were examined with Geneious v6.0.4 [50], aligned with Bioedit Sequence Alignment Editor [51] and compared with GenBank database (www.ncbi.nlm.nih.gov/BLAST). Phylogenetic analyses were performed using 25 sequences and 299 bp of the *16S rRNA* gene, and 39 sequences and 483 bp of the *18S rRNA* gene. For the combination data set (16S rDNA + 18S rDNA) we used 11 sequences and 764 bp. Two outgroups from the class Pycnogonida were used in order to root the trees: *Achelia hispida* (n: FJ862845) and *Ammothea* sp. (n: FJ862841) for the 16S rDNA tree, and *Achelia echinata* (n: AF005438) and *Callipallene* sp. (n: AF005439) for the 18S rDNA tree.

We used the Bayesian inference program MrBayes v3.2.5 [52] to estimate the phylogenetic trees. MrBayes uses Markov chain Monte Carlo (MCMC) methods to estimate the posterior distribution of model parameters. MODELTEST 3.7 [53,54] was used to select the best evolutionary model among 56 models of evolution by the Akaike Information Criterion. MCMC sampling was performed with 1,000,000 iterations using by default a burn-in of 25%. Bootstrap results are shown as a measures of tree repeatability and accuracy [55]. We then used the program FigTree to display the phylogenies (http://tree.bio.ed.ac.uk/software/figtree/).

## Results

### Mite prevalence in wolves, bats and marmots using qPCR for the *16S rRNA* gene

We screened for mite DNA in hair samples from three social mammal populations using the *16S rRNA* gene. We only considered qPCR samples to be positive when the melting curves (Tm) were close to the *Demodex* control value (Tm = 72°C± 2°C) and successfully sequenced. Samples were considered to be uncertain when the Tm was close to the *Demodex* control value but the sample was unseccusfully sequenced. The amplification cycle for *Demodex* qPCR control was on average Cp = 25 cycles. Three out of 22 wolf samples analyzed (14%) were uncertain. These three samples were from Mexican wolves and showed an average Tm = 72°C and Cp = 39 cycles. The most common skin location was the foot (N = 3), and one individual was also uncertain in the pre-auricular facial area. We could not use any mite sequence from wolves for further analyses since we obtained double PCR sequence products from each sample.

Five out of 28 bat samples (17%) were positive by qPCR and one was uncertain (Table 1). Three samples from *M*. *yumanensis* showed on average a Tm = 71°C and Cp = 36 cycles. A sample from *C*. *townsendii* showed a Tm = 72°C and Cp = 39 cycles. A sample from *M*. *californicus* dissociated at Tm = 71°C and Cp = 35 cycles. Finally, one *M*. *yumanensis* sample, unsuccessfully sequenced, dissociated at Tm = 73°C and Cp = 39 cycles.

Two out of 16 marmot samples (13%) were positive by qPCR and two were uncertain. Both positive samples showed a Tm = 73°C and Cp = 39 cycles. The other two uncertain samples showed a Tm = 72°C and 74°C and Cp = 39 cycles, respectively.

### 16S rDNA sequence variability

The mitochondrial 16S rDNA mite fragment from five bats, two marmots, thirteen dogs, four cats, one human and one ferret were successfully sequenced (Table 1 and S1 Table).

None of the mite sequences from bats and marmots were identical to any GenBank sequence previously submitted, including *Demodex* sequences (Table 1). The sequence called mite_bat1 was found in three different individuals and was submitted to GenBank (n: KT259444). Sequences named mite_bat2, mite_bat3, mite_marmot1 and mite_marmot2 were each found in one individual and submitted to GenBank (n: KT259445 and n: KU253782, n: KT259446 and KT259447, respectively). Mite sequences from seven dogs and the ferret were identical to the sequence of a *D*. *canis* obtained in dogs from Spain (Genbank accession number (n): JX390978). As no sequences from *Demodex* mites isolated from ferrets had been reported so far, we deposited the sequence (coverage and identity of 99%) in GenBank (n: KU253781). One sequence (named *D*. *canis1*) corresponded to *D*. *canis* found in dogs from China (n: JF784000) and one corresponded to *D*. *canis* variant *cornei* described in dogs from Spain (n: JX390979). Moreover, we found and submitted a new variant of *D*. *canis* (named dog3) in a dog sample from Rhode Island, US (n: KT259448), which had 98% identity with *D*. *canis* mites found in a Tibetan mastiff from China (n: JF784001). The last three mite sequences from dogs were identical to *D*. *injai* found in dogs from Italy and Spain (n: JX390980). Because one of our *D*. *injai* sequences was larger than the published sequences, we submitted it to GenBank (n: KT259449). The samples from the four cats corresponded to sequences published previously in GenBank: *D*. *cati* (2 samples), n: JX193759; *D*. *gatoi*, n: KF052996 and *D*. *felis*, n: KF052995. Finally, the *D*. *folliculorum* sequence was identical to the sequence of four Chinese *D*. *folliculorum* samples (n: JF783995, JF83996, FN42425 and FN42426).

In total, we aligned 25 16S rDNA fragments of 264 bp, including the 13 new sequences and 12 sequences—belonging to the Prostigmata (Trombidiformes) order—retrieved from GenBank (S1 Fig). Twenty-five different haplotypes were identified (14 *Demodex* mites and a further 11 Prostigmata mites) with nucleotide diversity π = 0.29, the number of polymorphic sites *S* = 138 and 261 total mutations. The nucleotide variability among *D*. *canis* (N = 5) was π = 0.02, *D*. *folliculorum* (N = 4) π = 0.02, and *Demodex* spp in cats (N = 3) π = 0.25. Table in S2 Table shows the overall percentage of adenine (A), cytosine (C), guanine (G) and thymine (T) nucleotides across all samples. The average nucleotide frequencies, excluding gaps, were 32.74% (A), 41.39% (T), 7.41% (C), and 18.46% (G). A bias toward A and T (74.13%; X^2^(24, *N* = 25) = 216, *p*<0.001) was consistent with the nucleotide composition of the mitochondrial DNA among Arthropoda phylum [12,56,57]. The distribution of the K2P distance for the *16S rRNA* gene was wide, ranging from 0.004 to 0.828 (S2 Fig). The highest K2P distance was found between samples *Diptilomiopus* sp. (Eupodina suborder) and mite_marmot1 (Prostigmata order), where the number of nucleotide differences was 102 (up to 264 positions). The lowest K2P distance (one nucleotide substitution) was between *D*. *canis* variants, namely: *D*. *canis* (UAB) versus *D*. *canis* (1) and *D*. *canis (cornei)* versus *D*. *canis* (2).

### 18S rDNA sequence variability

The nuclear 18S rDNA mite fragment from five bats, thirteen dogs, four cats, one human and one ferret were successfully sequenced (Table 1 and S1 Table). Unfortunately, we could not obtain the *18S rRNA* mite sequences from the two marmot samples.

We obtained ten different sequences (prefix UAB) and submitted nine to GenBank: sample mite_bat1 (N = 3), n: KU253783; mite_bat2, n: KU253784; mite_bat3, n: KU253785; *D*. *canis* (N = 10, including the two 16S rDNA-variants *cornei* and dog3), n: KU253790; *D*. *canis* (from the ferret sample) n: KU253791; *D*. *injai* (N = 3), n: KU253789; *D*. *cati* (N = 2), n: KU253788; *D*. *gatoi*, n: KU253786; *D*. *felis*, n: KU253787. The *D*. *folliculorum* sequence from one of the Spanish co-authors was identical to four obtained from American people and one obtained from a Chinese person (n: KF745889, KF745888, KF745886, KF745881, EU861211 and HQ728000).

In total, we aligned 39 18S rDNA fragments of 469 bp, including nine of our sequences (the sequence from the ferret and the sequences from the ten dogs were the same, named *D*. *canis* (UAB)) and 30 sequences belonging to the orders Prostigmata (Trombidiformes; 28) and Sarcoptiformes (two) were retrieved from GenBank (S3 Fig). We identified 39 different haplotypes (twenty-five were *Demodex* mites), with nucleotide diversity π = 0.1, the number of polymorphic sites S: 178 and 257 total mutations. The nucleotide diversity among *D*. *canis* (N = 5) was π = 0.04, *D*. *folliculorum* (N = 5) π = 0.02, *D*. *brevis* (N = 9) π = 0.01, and *Demodex* spp in cats (N = 3) π = 0.03.

The nucleotide frequencies were 29.02% (A), 29.61% (T), 17.59% (C), and 23.79% (G) (S3 Table). No bias toward A and T (58.63%; χ^2^(38, *N* = 39) = 29, *p* = 0.83) was observed among Acari sequences for this nuclear gene. The distribution of K2P distance for the *18S rRNA* gene was narrower than for the *16S rRNA* gene, ranging from 0.002 to 0.301 (S4 Fig). Interestingly, the highest K2P distances were found between *Agistemus* sp versus *D*. *cati* (115 nucleotide substitutions) and *Syringophilidae* sp (110 nucleotide substitutions), the three species belong of the Anystina suborder. The lowest K2P distances (one nucleotide substitution) were between species from the *Demodex* genus: *D*. folliculorum (127) versus *D*. *folliculorum* (UAB), *D*. *canis* (Dc2) versus *D*. *canis* (UAB) and *D*. *brevis* (141_3) versus *D*. *brevis* (127_5).

### Phylogenetic relationships within Prostigmata mites

**Fig 1:**
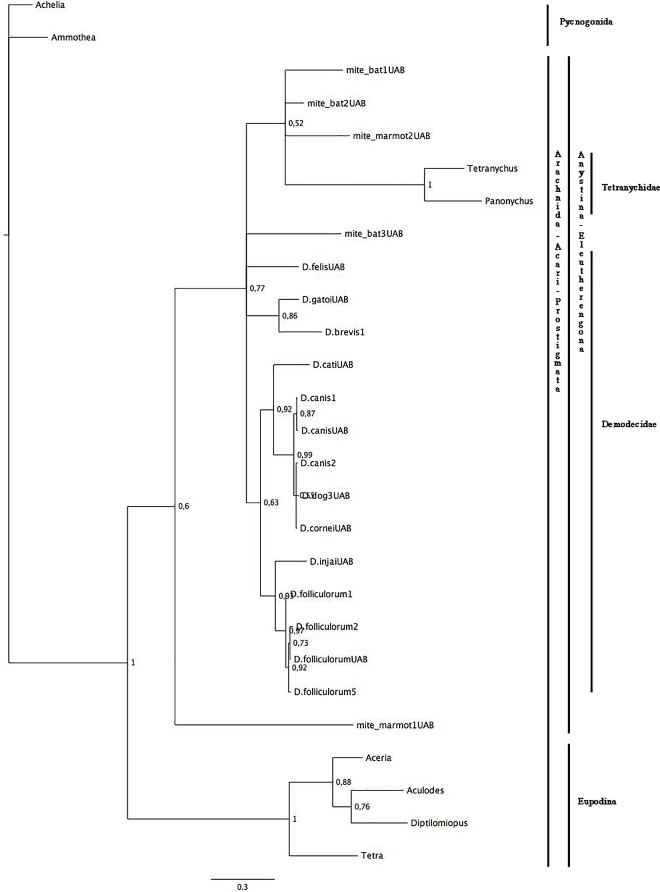
Phylogenetic analyses of Acari. The tree was estimated using MrBayes based on aligned fragments of the 16S rDNAgene. Branch support is based on 10,000 boostrap replications. The scale at the bottom measures genetic distances in nucleotide substitutions per site.

**Fig 2:**
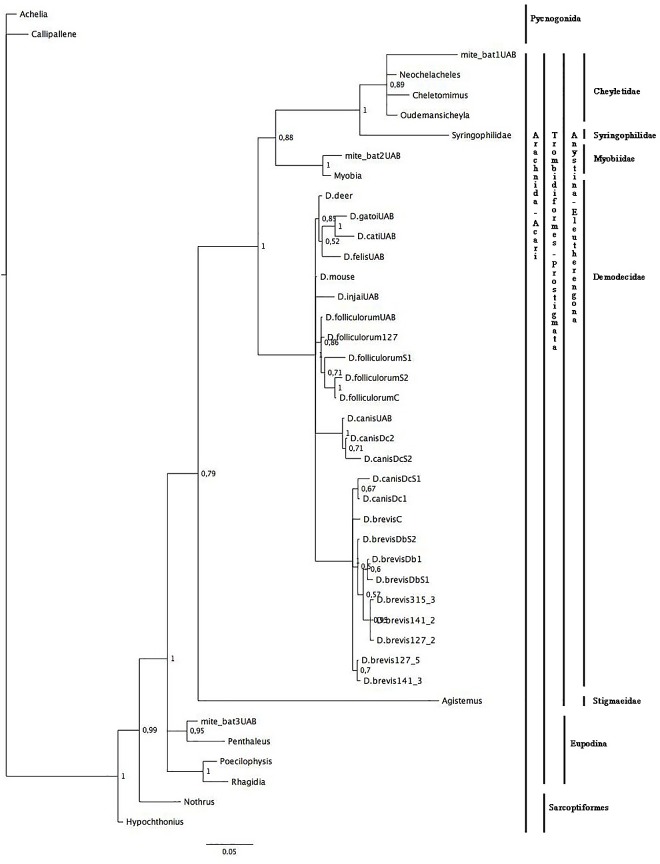
Phylogenetic analyses of Acari. The three was estimated using MrBayes based on aligned fragments of the 18S rDNA gene. Branch support is based on 10,000 boostrap replications. The scale at the bottom measures genetic distances in nucleotide substitutions per site.

**Fig 3:**
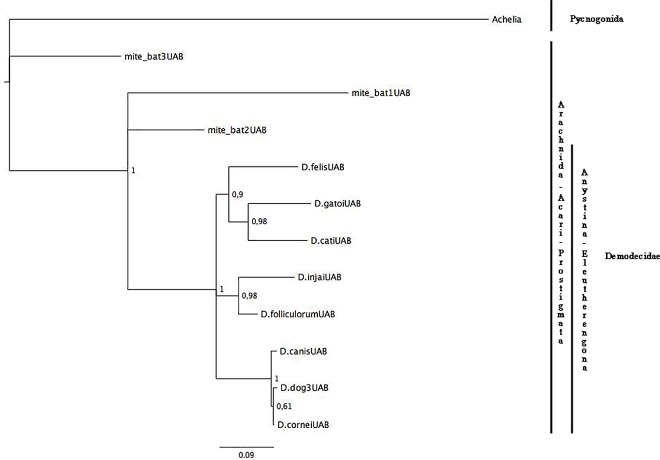
Phylogenetic analyses of Acari. The tree was estimated using MrBayes based on aligned fragments of combinated 16S rDNA and 18S rDNA genes. Branch support is based on 10,000 boostrap replications. The scale at the bottom measures genetic distances in nucleotide substitutions per site.

For the phylogenetic analyses, we used a 299 bp 16S rDNA fragment and a 483 bp 18S rDNA fragment, which include the regions of highest variability [58,59]. For the combination data set (16S rDNA + 18S rDNA), we used 11 sequences and 764 bp. All the trees were rooted using outgroups from the Pycnogonida class (Figs 1, 2 and 3). The transition (TIM+I+G) and the general-time-reversible (GTR+G) models were selected as the best-fitting models for *16S rRNA* and *18S rRNA* genes respectively, and for the combination data set, the Hasegawa-Kishino-Yano (HKY+G)(*16S rRNA*) and the Tamura-Nei (TrN+G)(*18S rRNA*) models were selected; (G: gamma distribution; I: proportion of invariable sites; for model details see Posada and Crandall 1998).

The 16S-Bayesian tree (Fig 1) showed a major split between Eupodina and Anystina suborders with bootstrap values >70%, suggesting that all of our samples belong to the Anystina cohort. The second split separated the sample mite_marmot1 from the rest of the samples, suggesting that it does not belong to the Demodecidae or Tetranychidae families. However, due to the low bootstrap value of <70%, this split needs to be evaluated with caution. The third split showed a bootstrap value >70%, but the taxa did not group according to host, namely: 1) samples mite_bat1 and mite_bat2 clustered with sample mite_marmot2 and the Tetranychoidea clade, but not with sample mite_bat3; 2) *D*. *gatoi* (host: cat) and *D*. *brevis* (host: human) were sister taxa (boostrap value >70%); 3) *D*. *cati* (host: cat) and *D*. *canis* clade (host: dog) were sister taxa (boostrap value >70%); and finally 4) *D*. *injai* (host: dog) and *D*. *folliculorum* clade (host: human) were sister taxa (boostrap value >70%).

The 18S-Bayesian tree (Fig 2) showed a clear split (bootstrap value 99%) between the Sarcoptiformes and the Trombidiformes (Prostigmata) mites. Subsequently, Prostigmata split in two clusters: Eupodina and Anystina with bootstrap values >90%. The mite_bat3 sample fell within the Eupodina cluster and was sister to *Penthaleus* sp. On the other hand, Anystina clustered in five mite families with boostrap values >70%. The deepest split within the Anystina cohort was from the Stigmaeidae family. The mite_bat1 sample appeared to be a member of the family Cheyletidae with Syringophilidae as the sister group, while the mite_bat2 sample appeared to be a member of the Myobiidae family, since it was a sister group to *Myobia* sp. The monophyletic Demodecidae family included all the *Demodex* spp. Four large clusters constituted this family: 1) the *D*. *canis* cluster; 2) the *D*. *folliculorum* cluster; 3) the *D*. *gatoi*, *D*. *cati* and *D*. *felis* cluster (host: cat) (in contrast to the 16S-Bayesian tree), and 4) the *D*. *brevis* cluster (host: human) with two *D*. *canis* sequences (host: dog).

Due to the limited availability, we only used UAB samples and an outgroup (*Achelia* sp.) to estimate the 16S+18S Bayesian tree (Fig 3). As the 18S Bayesian tree showed, mite sequences from bats were not included in the Demodecidae family. The deepest split in the tree separated mite_bat3. Mite_bat1 and mite_bat2 were sisters to the monophyletic *Demodex* cluster. Paradoxically, mite_bat1 and mite_bat3 were collected in close proximity (San Juan County, WA), whereas mite_bat2 was collected over 400 km away (eastern WA). On the other hand, *Demodex* spp. from cats clustered together, all the three *D*. *canis* variants (*canis*, *cornei*, dog3) were assigned to the same clade and *D*. *injai* and *D*. *folliculorum* formed a sister group. Bootstrap values over 90% in most of the nodes show the robustness of the tree.

### Sequence analysis: Divergence and interspecific genetic distances between and within Prostigmata mites

The phylogenetic trees did not cluster samples according to host species (Figs 1, 2 and 3). The K2P distances within groups were larger using the *16S rRNA* gene (from 0.238 to 0.432) than the *18Sr RNA* gene (from 0.025 to 0.068) (S2 and S4 Figs). For instance, within the family Demodecidae, 16S rDNA-K2P = 0.238 (including 14 sequences without sample mite_bat3) and 18S rDNA-K2P = 0.04 (including 25 sequences without sample mite_bat3). In the same way, the Eupodina cohort showed a 16S rDNA-K2P = 0.342 (four sequences) and an 18S rDNA-K2P = 0.069 (including three sequences and sample mite_bat3). The largest 16S rDNA-K2P distance (0.43) was observed within the Tetranychidae family, where samples from mite_bat1, mite_bat2 and mite_marmot2 were included. These results may suggest that these three samples belong to the Eleutherengona cohort, but not to the Tetranychidae family. In fact, according to the *18S rRNA* gene (Fig 2), sample mite_bat1 belongs to the Cheyletidae family (N = 4; π = 0.07; K2P = 0.06), sample mite_bat2 belongs to the family Myobiidae (N = 2; π = 0.03; K2P = 0.03) and sample mite_bat3 belongs to the suborder Eupodina (N = 4; π_3_ = 0.05; K2P = 0.07). Unfortunately, we could not obtain mite sequences from marmots using the *18S rRNA* gene. However, we may assume that the two marmot samples belong to the Anystina suborder since the greatest distance between groups was between the Eupodina cohort and sample mite_marmot1 (K2P = 0.74).

## Discussion

### Mite detection and prevalence in social mammals

We detected the presence of mite DNA in bats (17%) and marmots (12%) through real-time qPCR. The presence of mite DNA in wolves (14%) was uncertain since we obtained qPCR dissociation curves but not mite DNA sequences. Therefore, we recommend to carry out ethanol precipitation during DNA extraction to obtain higher mite DNA quality, and/or designed more specific PCR primers to obtain clear mite sequences from wolf samples. Identifying mites in wolves would help to elucidate whether mite evolution paralleled dog domestication about 15,000 years ago. In contrast, despite only sampling one site on the body, the high mite prevalence in bats and marmots may indicate that mites are common inhabitants of their skin.

### 16S rDNA and 18S rDNA sequence variability

This research was conceived to detect mites in social mammals by using specific primers that successfully amplified *Demodex* clinical samples. We found that bats and marmots do not harbor *Demodex* mites, at least at the limited sites that were sampled; instead, we detected mites from the Prostigmata order. The 16S rDNA-K2P distance between member of the *Demodex* genus (S2 Fig) ranged between 0.004 and 0.404 (average = 0.24). The 16S rDNA K2P-distance among the three bat samples and between the two marmot samples was on average 0.389 and 0.427, respectively, about two-fold greater than within the genus *Demodex*. Therefore, mites from bats and marmots cannot be assigned to the same genus based only on a common host. The lack of information in the GenBank database regarding Acari 16S rDNA sequences did not allow for the identification of these five mites. For that reason, we decided to sequence a fragment of the nuclear *18S rRNA* gene, since more of these sequences are available in the GenBank database. Using this method, we found that 18S rDNA-K2P distances <7% between mite_bat1 and the family Cheyletidae, between mite_bat2 and the family Myobiidae, and between mite_bat3 and the suborder Eupodina. In general, the genetic distances among species (interspecific) are larger than intraspecific distances. Small genetic distances indicate that they are closely related and share a recent common ancestor. These results are consistent with previous research showing that intraspecific distances range from 0 to 0.05 and interspecific distances from 0.02 to 0.2 [18,60–62]. Therefore, we can conclude that samples from bats do not belong to the *Demodex* genus, but rather to the genera *Neuchelacheles* (mite_bat1), *Myobia* (mite_bat2) or *Penthaleus* (mite_bat3); these are all Prostigmata mites. We were unable to obtain 18S rDNA mite sequences in marmots; however, we can conclude that the mites found in marmots (as in bats) are not *Demodex* spp based on 16S rDNA sequences even though we used primers originally designed to detect *Demodex* mites.

Genetic variability values showed that the nuclear *18S rRNA* gene is more conserved than the mitochondrial *16S rRNA* gene. Nucleotide diversity, polymorphic sites and number of mutations were about two-fold greater for the 16S than the *18S rRNA* gene. We recommend using the 18S rDNA fragment to identify mites for the classification of distantly related members of the Prostigmata order, and the 16S rDNA to discriminate at lower taxonomic levels, for instance among members of the *Demodex* genus [14,20,33–35].

### *Demodex* mite variability and distribution

The nucleotide diversity using the *16S rRNA* gene (π = 0.2) among members of the *Demodex* genus was five-fold greater than for the *18S rRNA* gene (π = 0.04), showing again that it is better to use the *16S rRNA* gene to discriminate at lower taxonomic levels. The nucleotide diversity (*π)* within *D*. *canis*, *D*. *folliculorum* and *D*. *brevis* was low and similar using both the 16S and *18S rRNA* genes. However, among our samples (Table 1 and S1 Table), four variants of *D*. *canis* (UAB, 1, *cornei* and dog3) were differentiated using the *16S rRNA* gene, but only one using the *18S rRNA* gene (*D*. *canis* (UAB)). Our *D*. *canis* (UAB) sequences from dogs and a ferret were from Austria, Spain, the eastern Caribbean and the USA, and identical (except for one nucleotide) to a dog from Georgia (USA) [19]. Zhao et al. [20,63] found two and six *D*. *canis* variants using the *16S rRNA* and *18S rRNA* genes respectively, in dogs from China. Therefore, we can conclude that the distribution of *D*. *canis* (UAB) is wide, occurring in both the Old and the New Worlds. We found similar results for *D*. *injai* (host: dog) and *D*. *folliculorum* (host: human), which are distributed across Europe, Asia (for *D*. *folliculorum*) and the USA. Among *Demodex* mites in cats, we found significant π value differences between both genes. Nucleotide diversity was almost nine-fold greater using *16S rRNA* than *18S rRNA*. Paradoxically, despite having the highest π value, the three cats analyzed were exclusively from Europe. Additional cats should be analyzed throughout the world to better understand their mite variability and distribution. *Demodex* mites in cats were assigned differently according to the gene used. While the *18S rRNA* gene and the combination data (16S rDNA + 18S rDNA) clustered *D*. *gatoi*, *D*. *cati* and *D*. *felis* according to their host (cat), the *16S rRNA* gene clustered *Demodex* mites in cats with dogs and humans. Ferreira et al. [5] and Frank et al. [14] found similar results using 16S rDNA. There are no previously published results for cats using the *18S rRNA* gene. *D*. *brevis* was also clustered differently according to *16S rRNA* and *18S rRNA* genes, and was closely related to *D*. *gatoi* (in agreement with Frank et al. [14]) and to *D*. *canis* (in agreement with Thoemmes et al. [22]). In the same way, *D*. *injai* was more closely related to *D*. *folliculorum* instead of *D*. *canis*. As mites in bats and marmots, some *Demodex* mites did not consistently cluster according to host species.

### *Demodex, Myobia, Neochelacheles* and *Penthaleus* mites as potential parasites

The phylogenetic structure revealed by the Mr. Bayes analysis of 16S rDNA and 18S rDNA the new mite sequences (prefix UAB) all belong to members of the Prostigmata. Except for samples mite_bat3 and mite_marmot1, all our samples appeared to be members of the Anystina suborder, where most of the mites are known predators or parasites [36]. For instance, *Demodex* mite overgrowth causes severe and prevalent dermatitis in the host. Demodicosis has been extensively reported in domestic pets and even in humans [1,7,8,64]. *Myobia* sp is another mite species that infests marsupials, insectivores, bats and rodents [34,65], and *Neochelacheles* sp has been found in North America and the Philippines where it parasitizes several Tenebrionid beetles [66]. Sample mite_bat3 appeared to be a member of the Eupodina suborder, specifically the *Penthaleus* genus, considered a major agricultural pest in many temperate areas of the world, including the five continents [67]. Carrier wild hosts often go undiagnosed, allowing infestations to spread. Here, we have demonstrated an effective and easy molecular method to detect potential parasites in wild animals that, without control, can cause pest and disease.

### Parasite-host specificity of skin Prostigmata mites?

Although mites are host-specific and usually do not cross-infest, transmission between individuals within the same colony may occur during allogrooming, breeding, fighting, aggregating, or from mother to offspring during birth or nursing [21,68–71]. Transmission does not necessarily result in an individual developing a disease (e.g. demodicosis), as this may occur only in the case of an underlying immune defect. In our study, there were no known diseases in the population at the time. Bats live in colonies ranging from a few dozen to hundreds of thousands of individuals [45,46], while marmot colonies can reach up to thirty individuals [43]. The high variability (one species carrying two different families of mites) in marmots may be partially explained by the fact that the two positive mite samples were taken from two different colonies that are behaviorally and geographically isolated from one another (Table 1). In fact, three positive samples (mite_marmot1 and the two unsuccessfully sequenced samples) were located on the east side of the East River and mite_marmot2 on the west side. More comprehensive surveys within this population could elucidate the patterns of social transmission and show whether mite speciation occurs according to colony rather than host species.

Regarding bats, we found three different mites in three different species of bats. Interestingly, the *Myotis* genus harbors two closely related mites (*Neuchelacheles* sp. and *Myobia* sp.) despite being collected over 400 km away from one another (Douglas County versus San Juan County, WA, USA) (Table 1). In contrast, *Penthaleus* mites from *C*. *townsendii* were collected at the same site and on the same day as *Neuchelacheles* mites (Douglas County). In this case, the geographic distance is negligible while the phylogenetic relationship is significant, since these mites belong to two different suborders (Anystina versus Eupodina). Therefore, it seems that each bat species harbors a mite that has evolved according to host species. Moreover, two *M*. *yumanensis* bats harbored identical *Neuchelacheles* mites, indicating feasible interspecific cross-infection at site six. Our results do not match with the results from other authors. Desch [72] described morphologically (but not genetically) a new species of hair follicle mite (*Demodex nycticeii*) in the evening bat (*Nycticeius humeralis*), and Lankton et al. [16] found a 99.6% homology to *D*. *canis* using the same *16S rRNA* gene among four captive wild-caught big brown bats (*Eptesicus fuscus*) in eastern Tennessee (USA). Our bats were also captured in the USA and belong to the same family, *Vespertilionidae*, but not to the same genus. An additional difference was the captive status of Lankton’s bats.

Therefore, one question arises here: does captivity imply the transmission of *Demodex* in mammals, namely *D*. *canis*? Recently, Izdebska et al. [15] found *Demodex* mites morphologically similar to *D*. *canis* in otters (*Lutra lutra*). Interestingly, the sample from the ferret, a Mustelidae like the otter, was morphologically and genetically identical to *D*. *canis*. *D*. *canis* also have been found in cats [5,15,16]. All these mammals are directly related to human activities (e.g. domestication, captivity, hunting, furs). Furthermore, the monophyletic *Demodex* clade shows closely related dog and human *Demodex* sequences (Fig 2). Therefore, *D*. *canis* mite evolution may be a consequence of the relationship between “human-related” mammals and humans. Thoemmes et al. [22] concluded in their study that *D*. *brevis* may have colonized humans from wolves during their domestication. However, it could also be feasible that *D*. *canis* may have colonized dogs from humans after their domestication. Additional hair samples from wolves and mammals related to human activities and mite genes (e.g mtDNA COI or rDNA ITS2) should be analyzed to elucidate the evolution, adaptability and transmission of *D*. *canis* mites.

## Supporting Information

S1 Fig 16S rDNA fragment alignments. (TIF)

S2 Fig Matrix of sequence distance (lower left diagonal) and absolute nucleotide differences (upper right diagonal) on pairwise comparisons of the *16S rRNA* gene among thenty-five Prostigmata species. Samples with the prefix UAB are those sequenced in the present study. Those in bold are new sequences. The rest are sequences retrieved from GenBank. Sequence distances were estimated using the Kimura 2-parameter model.
(TIF)

S3 Fig 18S rDNA fragment alignments. (TIF)

S4 Fig Matrix of sequence distance (lower left diagonal) and absolute nucleotide differences (upper right diagonal) based on pairwise comparisons of the *18S rRNA* gene among thirty-nine Acari species. Samples with the prefix UAB are those sequenced from the present study. Those in bold are new sequences. The rest are sequences retrieved from GenBank. Analyses were conducted using the Kimura 2-parameter model.
(TIF)

S1 Table Source of *Demodex* mites sequenced in the study. (XLSX)

S2 Table Nucleotide percentage in Prostigmata mites using the *16S rRNA* gene. Samples with the prefix UAB are those sequenced from the present study.
(XLS)

S3 Table Nucleotide percentage in Acari mites using the *18S rRNA* gene. Samples with the prefix UAB are those sequenced from the present study.
(XLS)
