# Supplementary figures and images for: Detection, Prevalence and Phylogenetic Relationships of Demodex spp and further Skin Prostigmata Mites (Acari, Arachnida) in Wild and Domestic Mammals
Source: PLoS One. 2016 Nov 1;11(11):e0165765. doi: 10.1371/journal.pone.0165765 (PMC5089713; doi:10.1371/journal.pone.0165765)

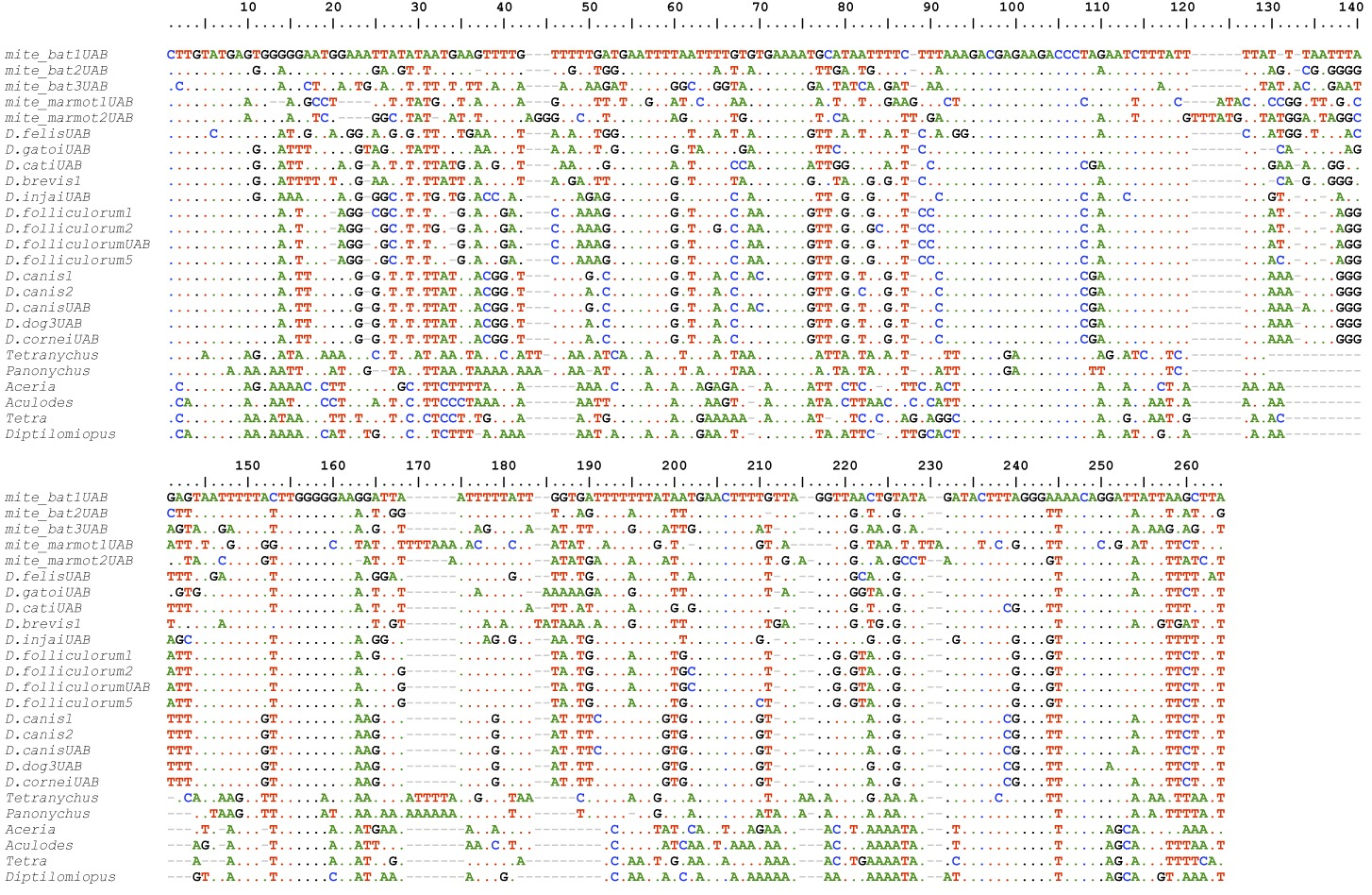

Supplement: S1 Fig — (TIF) [file pone.0165765.s001.tif]

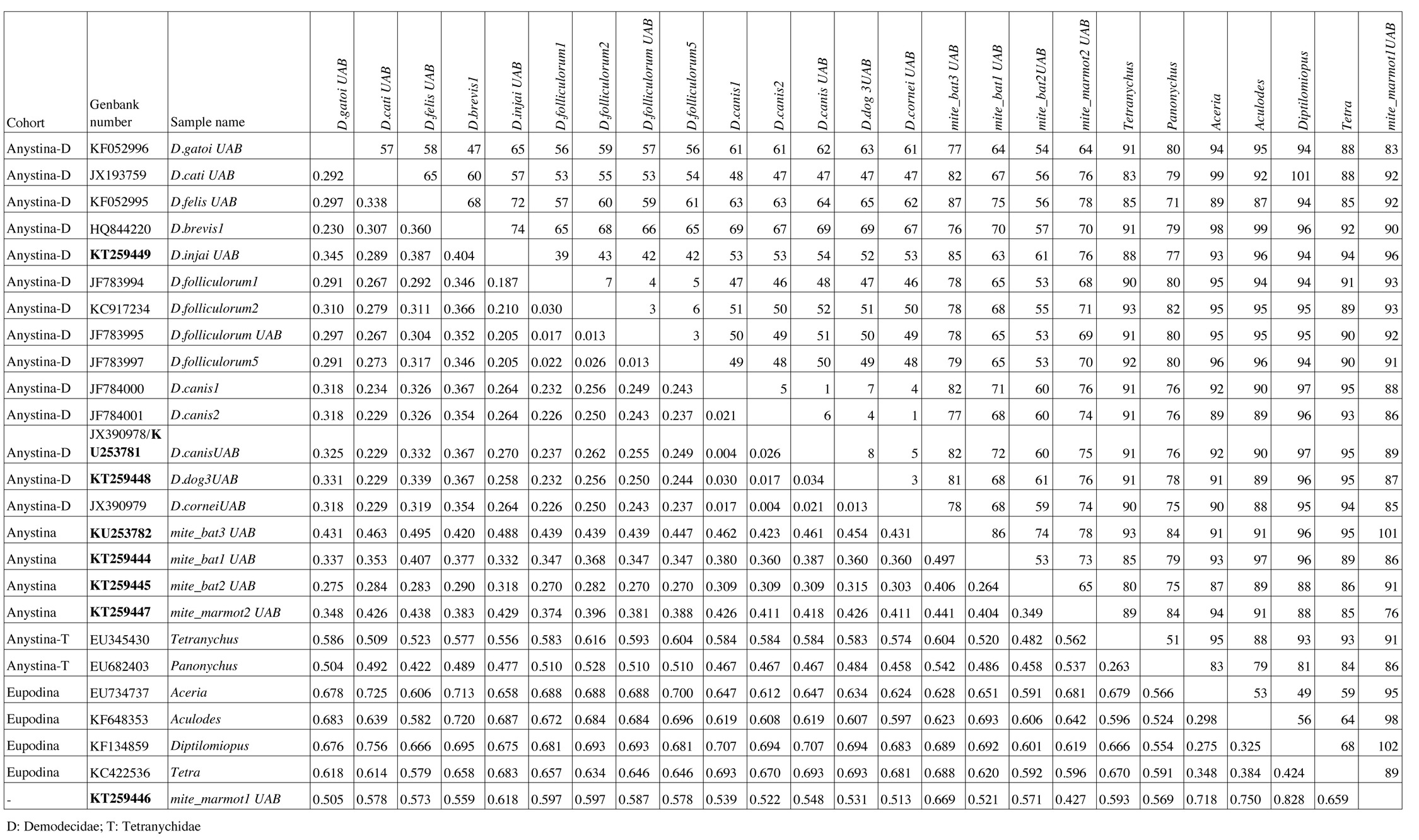

Supplement: S2 Fig — Samples with the prefix UAB are those sequenced in the present study. Those in bold are new sequences. The rest are sequences retrieved from GenBank. Sequence distances were estimated using the Kimura 2-parameter model. (TIF) [file pone.0165765.s002.tif]

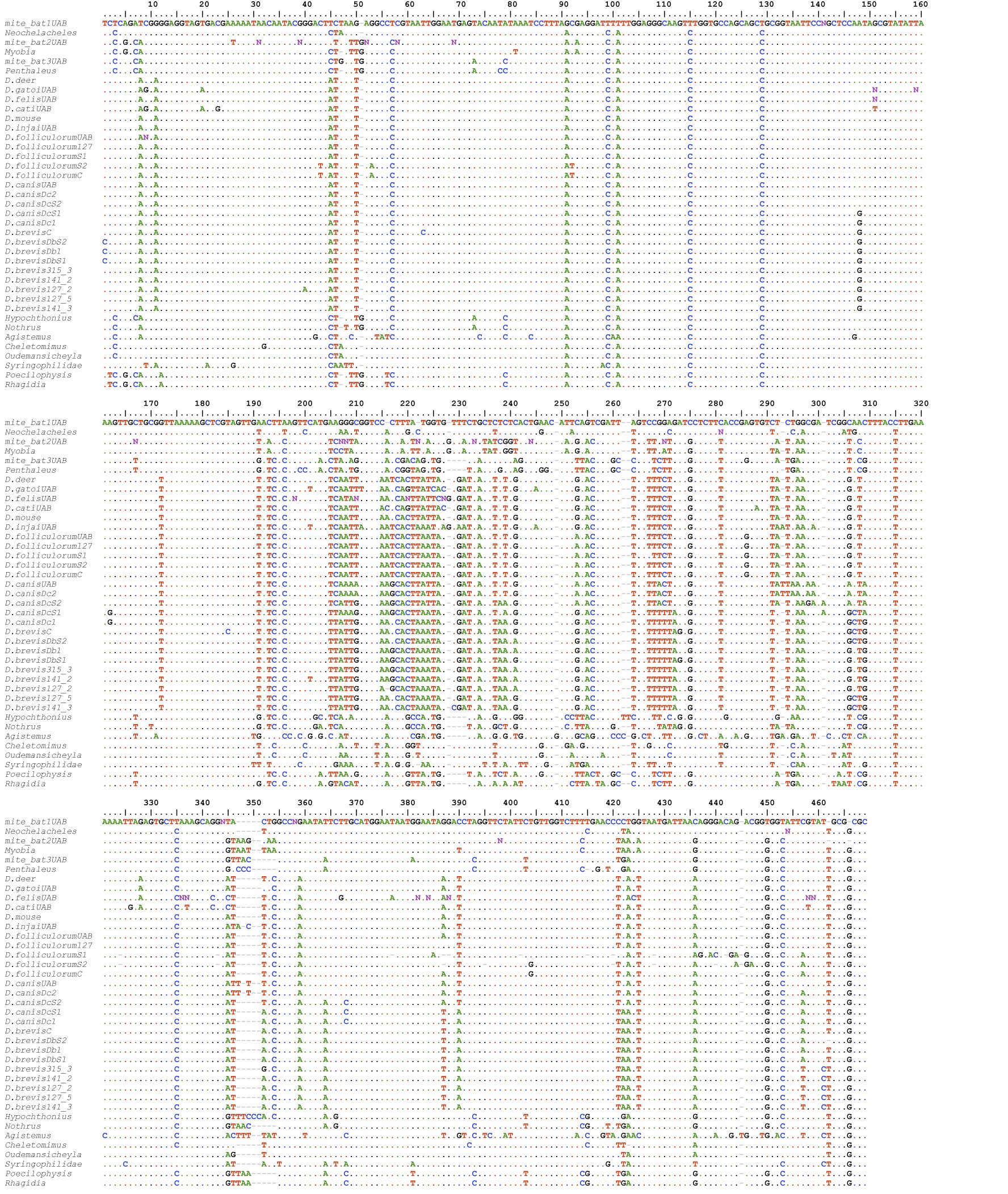

Supplement: S3 Fig — (TIF) [file pone.0165765.s003.tif]

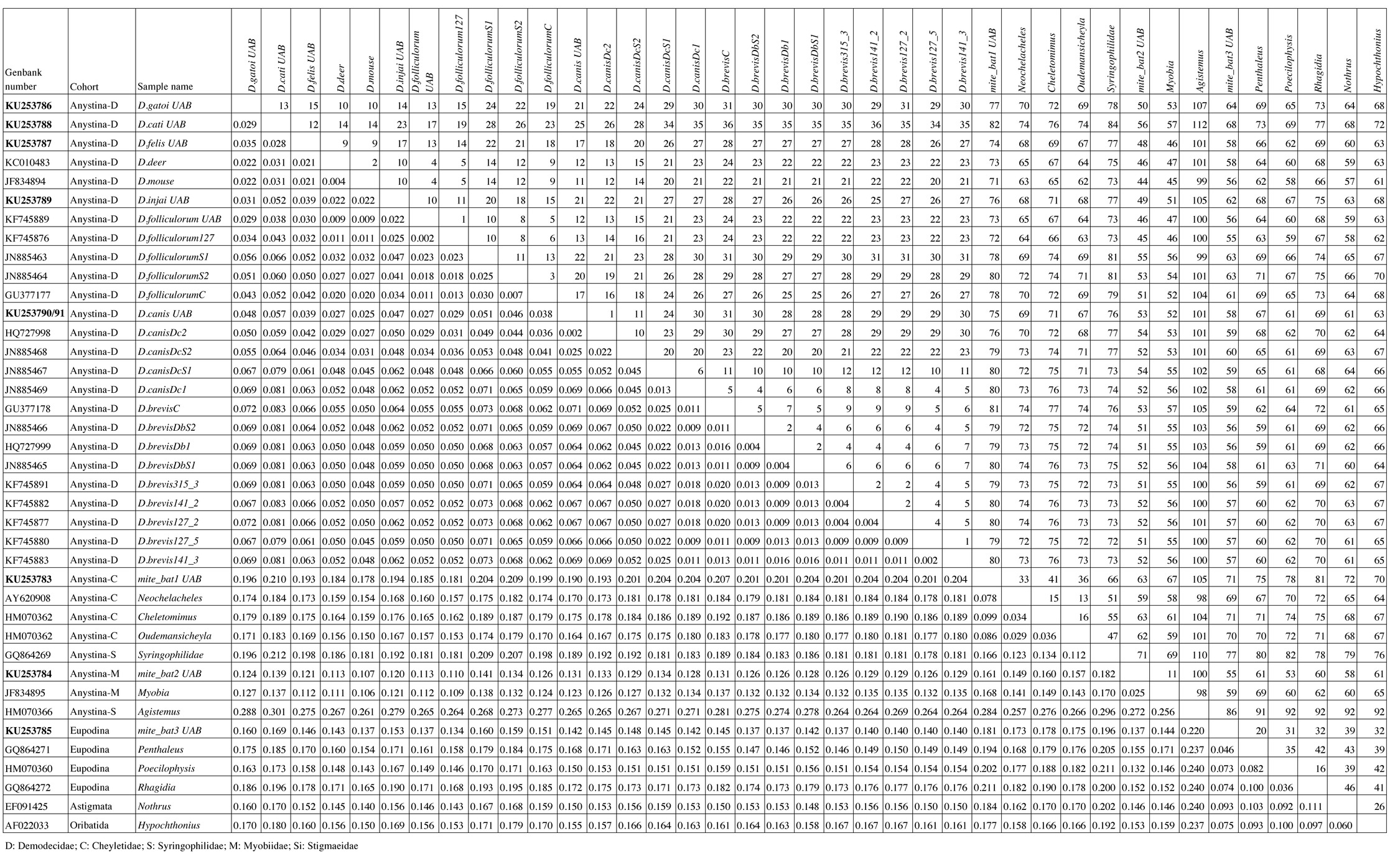

Supplement: S4 Fig — Samples with the prefix UAB are those sequenced from the present study. Those in bold are new sequences. The rest are sequences retrieved from GenBank. Analyses were conducted using the Kimura 2-parameter model. (TIF) [file pone.0165765.s004.tif]
